# Feasibility of detecting myocardial ischemia using first-pass contrast MRI and regadenoson

**DOI:** 10.1186/1532-429X-14-S1-P11

**Published:** 2012-02-01

**Authors:** Matthew Lyons, Cylen Javidan-Nejad, Ibrahim M Saeed, Donna Lesniak, Gary R McNeal, Agus Priatna, Robert J Gropler, Pamela K Woodard

**Affiliations:** 1 Metrohealth Medical Center, Cleveland, OH, USA; 2 Washington University School of Medicine, St. Louis, MO, USA; 3 St. Luke's Cardiovascular Consultants, Kansas City, MO, USA; 4 Siemens Medical Systems, Malvern, PA, USA

## Summary

A single injection of regadenoson can be used instead of an adenosine infusion to produce coronary vasodilatation and demonstrate myocardial ischemia during first-pass perfusion cardiac MRI.

## Background

Cardiac stress MR perfusion imaging requires an MRI compatible infusion pump for the administration of adenosine or a non-MRI compatible pump housed in the control room or beyond the 10-Gauss line. Regadenoson is a recently FDA-approved A2A receptor agonist that can be given intravenously in a single bolus. It has been shown to provide diagnostic information regarding myocardial ischemia on SPECT-MPI.

## Methods

42 patients (34 M, 55 yrs, range 41-73 yrs) with a reversible myocardial perfusion defect on SPECT-MPI underwent a cardiac perfusion MRI within 7 days of the SPECT-MPI. MR exams consisted of short and long axis cine steady state free precession (SSFP) imaging, matched gradient-recalled echo (GRE) GRAPPA temporal parallel acquisition (TPAT) first-pass stress perfusion (TR 2.3 msec, TE 1.1 msec, 80*256 matrix, 1.4 x 3.1mm2), and delayed contrast-enhanced (DCE) T1 GRE imaging. First-pass perfusion images were obtained 30 seconds after regadenoson 400 micrograms administered in a single IV bolus and during power injection of 0.075 mmol/Kg of gadobenate dimeglumine at 5 mL/sec IV followed by normal saline flush. DCE imaging was obtained 10 minutes after injection of an additional 0.025 mmol/Kg of contrast agent.

## Results

All but one patient tolerated the regadenoson MR examination. One patient had chest pain shortly after imaging, and received aminophylline, with resolution of symptoms. MR showed ischemia in 33/42 subjects. In 8 subjects the MR perfusion exam was normal. Five of these 8 patients underwent clinically-ordered invasive cardiac catheterization (ICA) within 3-18 days of the MRI examination. ICA showed no stenoses, suggesting SPECT attenuation artifact. The other 3/8 patients had no MACE within 30-180 days. In one patient, SPECT demonstrated ischemia only, while MRI showed infarct only in the same segment.

## Conclusions

Regadenoson can be used in cardiac MR perfusion imaging to demonstrate ischemia. MRI perfusion imaging may be useful in differentiating attenuation artifact from true disease.

## Funding

Funding for this project was provided by Astellas. Additional research support provided by Siemens Medical Systems.

